# The Role of Acrolein in Neurodegenerative Diseases and Its Protective Strategy

**DOI:** 10.3390/foods11203203

**Published:** 2022-10-14

**Authors:** Xinxin Chang, Yudan Wang, Bing Zheng, Yi Chen, Jianhua Xie, Yiming Song, Xiaomeng Ding, Xiaoyi Hu, Xiaobo Hu, Qiang Yu

**Affiliations:** State Key Laboratory of Food Science and Technology, China-Canada Joint Laboratory of Food Science and Technology (Nanchang), Key Laboratory of Bioactive Polysaccharides of Jiangxi Province, Nanchang University, 235 Nanjing East Road, Nanchang 330047, China

**Keywords:** acrolein, neurodegenerative diseases, oxidative stress, inflammation

## Abstract

Neurodegenerative diseases are characterized by a massive loss of specific neurons, which can be fatal. Acrolein, an omnipresent environmental pollutant, is classified as a priority control contaminant by the EPA. Evidence suggests that acrolein is a highly active unsaturated aldehyde related to many nervous system diseases. Therefore, numerous studies have been conducted to identify the function of acrolein in neurodegenerative diseases, such as ischemic stroke, AD, PD, and MS, and its exact regulatory mechanism. Acrolein is involved in neurodegenerative diseases mainly by elevating oxidative stress, polyamine metabolism, neuronal damage, and plasma ACR-PC levels, and decreasing urinary 3-HPMA and plasma GSH levels. At present, the protective mechanism of acrolein mainly focused on the use of antioxidant compounds. This review aimed to clarify the role of acrolein in the pathogenesis of four neurodegenerative diseases (ischemic stroke, AD, PD and MS), as well as protection strategies, and to propose future trends in the inhibition of acrolein toxicity through optimization of food thermal processing and exploration of natural products.

## 1. Introduction

Acrolein is a highly active α, β-unsaturated aldehyde which is a colorless, flammable, and volatile pure liquid known for its pungent odor and intense irritation of the mucous membrane, especially the eyes and upper respiratory tract [1]. Acrolein is known as an exogenous toxin produced by external environmental pollutants, including smoking, incomplete combustion of plastic materials, cooking, and smoke, and the exposure may be exceptionally high in smoking places and urban areas with a high volume of car traffic. In addition, acrolein was classified as a priority control contaminant by the US Environmental Protection Agency (EPA) (CASRN 107-02-8). When the concentration of acrolein in the air is approximately 0.25 ppm, humans can perceive it [2]. The primary source of human exposure to acrolein was incomplete burning of organic materials, estimated at 5 mg/day and fat or oil produced when cooking or frying food [3].

Neurodegenerative diseases are complex diseases that affect the living quality of patients, causing disability and even leading to death. Neurodegenerative diseases stemming from neuronal and/or myelin loss might worsen over time and lead to dysfunction [4]. Neurodegenerative diseases are generally classified into two categories: acute neurodegenerative diseases and chronic neurodegenerative diseases, the former mainly including stroke and brain injury, and the latter primarily including Parkinson’s disease (PD), Alzheimer’s disease (AD), etc. [5]. As one of the major hazards of the brain, ischemic stroke is an umbrella term for a wide range of diseases, including thrombosis, cerebral embolism, lacunar infarction, and transient ischemic attacks, the core of which is neurological dysfunction caused by neuronal damage [6]. AD is a degenerative disease of the central nervous system (CNS), and its causes and pathogenesis have not been elucidated. The characteristic pathological changes consist of extracellular senile plaques formed by beta-amyloid deposition, intracellular tangles of neurogenic fibers due to tau protein hyperphosphorylation, as well as a neuronal loss with glial cell hyperplasia. PD is common in the middle-aged and elderly population, which is associated with environmental and neurological ageing factors. The main pathological lesion is the degenerative death of dopaminergic neurons in the midbrain substantia nigra. The other neurodegenerative disease, Multiple Sclerosis (MS), is a putative autoimmune demyelinating disease for the CNS with multiple endogenous and exogenous factors [7]. The treatment of these diseases has been a challenge for many years.

The large accumulation of acrolein in the body, either by exogenous ingestion or by disease, may have adverse neurological effects, which has been confirmed in studies in vitro and in vivo. Therefore, it is quite vital to improve the understanding of acrolein in neurodegenerative diseases. In addition, many drugs and natural products have been reported to have the function of treating acrolein poisoning or inhibiting toxicity. However, the regulatory mechanism and protective strategies of exogenous acrolein in neurodegenerative diseases have not been systematically elucidated. Therefore, this review aimed to clarify the role of acrolein in the four neurodegenerative diseases (ischemic stroke, AD, PD and MS) as well as strategies for its protection and to propose future trends in the inhibition of acrolein toxicity through optimization of food thermal processing and exploration of natural products.

## 2. Research Methodology

We conducted a comprehensive literature search in four databases, including China Knowledge Network, Web of Science, Science Direct, and Google Scholar. Searches were conducted using the following medical terms: “acrolein” AND (“neurodegenerative diseases” OR “ischemic stroke”, “Alzheimer’s disease” OR “Parkinson’s disease” OR “Multiple sclerosis” OR “oxidative stress” OR “inflammation”) AND (“antioxidants” OR “acrolein scavengers”). In addition, we manually checked the reference lists of the selected papers to further refine the relevance of the topic. We searched a total of 1461 documents and filtered the most relevant articles by title and abstract. The search was conducted until July 2022, with no restrictions on language or publication date. All references were imported into EndNote 20 software.

## 3. Acrolein Sources and Toxicokinetics

### 3.1. Source of Acrolein

**Figure 1:**
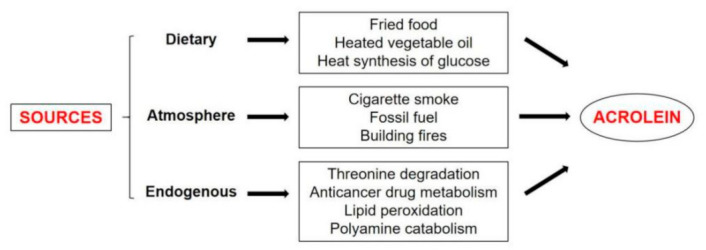
The sources of acrolein.

The main sources of acrolein are diet, atmosphere, and endogenous lipid peroxidation, as shown in Figure 1.

#### 3.1.1. Dietary

A working group of the World Health Organization (WHO) determined the acceptable daily intake (ADI) for acrolein, which was 7.5 μg per kg of a consumer’s body weight per day [8]. However, the maximum daily acrolein exposure in humans was much higher than the standard, approximately 0.1 mg/kg/day [9].

Researchers have recorded the elevation of acrolein and urinary metabolites in non-smoking Asian women who regularly cooked fried foods, which suggests that the intake of acrolein in the lungs comes from family sources encountered in daily life [3,9]. Since the last century, scientists have detected acrolein in cured pork, trevally, bread, grilled meat, and baked fish. Some foods such as bread, cheese, and alcoholic beverages contain much higher levels than the standard, as detailed in Table 1. One of the major pathways for acrolein production in food is glycerol dehydration, so acrolein can be detected in animal or vegetable fats at any time under high temperatures [10]. Acrolein was present in heated vegetable oils, and the amount of acrolein could be 10 times higher after reheating [11]. Heating oil could reduce the cis-double bond content of triglycerides and increase the formation of trans unsaturated aldehydes such as acrolein [12]. Furthermore, there were studies that identified the thermal synthesis of acrolein from glucose. Foods containing carbohydrates can form reactive carbohydrate intermediates when heated or baked. These intermediates underwent carbon–carbon cleavage or react with amino acid residues in proteins. According to research, glucose was dehydrated and alcohol-formaldehyde cleaved to first form hydroxyacetone, the precursor substance of acrolein, which was then dehydrated to form acrolein [1]. 

#### 3.1.2. Atmosphere

The main source of acrolein, as well as other atmospheric aldehydes, is the incomplete combustion of organic compounds. It is detected in a large number of different fumes from cigarettes, fossil fuels (such as gasoline or petroleum), synthetic polymers, building fires, food, plants, paraffin, animals, fats, vegetables, and trees [2]. The EPA of the United States reported that the primary source of acrolein exposure for the population in general was the atmosphere, where atmospheric levels varied from 8.2 μg/m^3^ to 24.6 μg/m^3^, representing 13% of total aldehydes in the ambient air. Air pollution due to vehicle exhaust and incomplete combustion of waste is an important source of acrolein in the atmosphere [13]. Besides, cigarette smoke was another influential source of acrolein exposure [14,15]. Exposure to acrolein from cigarette smoking is the highest, accounting for half or more of the overall human exposure to acrolein through all origins [1]. 3-HPMA was the main metabolite of acrolein, and estimates of acrolein production, based on measurement of urine HPMA, indicated that the urine of smokers contained 2–4 lM acrolein, while the urine of healthy adults of non-smokers contained 1–2 lM acrolein. Recent studies have shown that the shift from tobacco to electronic cigarettes greatly reduced exposure to toxic substances such as acrolein [16]. In addition, in vitro studies have demonstrated that acrolein in tobacco smoke was directly involved in the regulation of lung epithelial cells on key pro-inflammatory mediators, such as neutrophil attractant IL-8. 

#### 3.1.3. Endogenous Lipid Peroxidation

In addition to external intake, the body itself can produce an amount of acrolein. Acrolein could be produced in the following ways: (1) degradation of threonine in neutrophils by myeloperoxidase [17,18]; (2) metabolism of anticancer drugs such as cyclophosphamide; (3) lipid peroxidation of polyunsaturated fatty acids (LPO) to produce polyunsaturated fatty acids (PUFAs) [9,19]; and (4) catabolism of polyamines such as spermine and spermidine by amine oxidase [20]. It has been known that spermidine and spermine promoted the expression of phase 2 genes in cultured cells by producing acrolein and activating the NRF2-ARE pathway [21]. However, the production of acrolein from these precursors in cells has not been thoroughly studied.

### 3.2. Toxic Kinetics of Acrolein

Exogenous acrolein is absorbed by the body mainly through inhalation and oral administration, and there is limited information on its absorption through skin contact. Among them, inhalation is the most prevalent method of exposure. The majority of the absorbate remained in the respiratory tract tissues and was expelled from the respiratory system during exhalation, but sometimes part of it could be taken up into the bloodstream and distributed all over the body [2]. The long-term existence of acrolein in organs could be accounted by the reversible binding of acrolein to plasma proteins and hemoglobin, so acrolein might be released from protein adducts and distributed into tissues [22].

**Figure 2:**
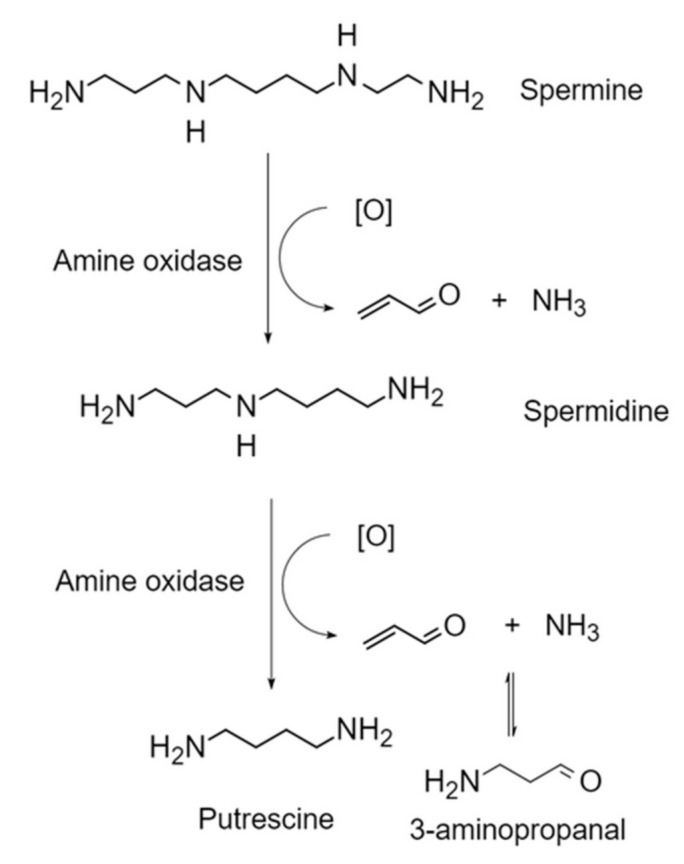
The pathway of acrolein production by spermine catabolism.

**Figure 3:**
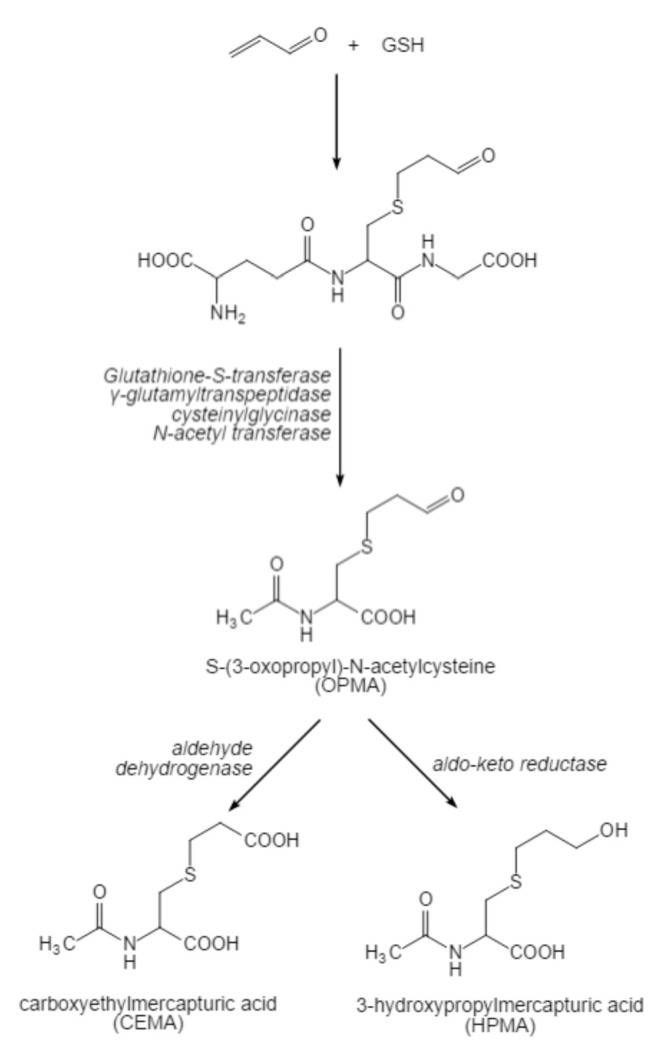
The metabolic process of acrolein.

Recently, it has been shown that endogenous acrolein is principally shaped by 3-aminopropionaldehyde (NH_2_[CH_2_]_2_CHO) efficiently produced by spermine oxidase (SMO) from spermine, while 3-acetylaminopropionaldehyde (CH_3_CONH[CH_2_]_2_CHO) produced by SSAT and acetyl polyamine oxidase (AcPAO) from spermine and spermidine is less efficient. The production of acrolein from 3-aminopropanal has been reported to be easier than from 3-acetylaminopropanal [23]. The pathway of acrolein production by spermine catabolism is shown in Figure 2. Glutathione (GSH) binding was the main detoxification pathway, which accounted for 60–70% of the total acrolein metabolism [1]. The reaction of the 2,3- double bond with glutathione crystal thiol was mainly carried out by the non-enzymatic pathway or glutathione chain enzyme catalysis [18]. After acrolein was bound to GSH, c-glutamic acid and glycine residues were cleaved, respectively, in the liver and kidney. The N-acetylation of the resulting cysteine conjugate produced S-(3-oxopropyl)-N-acetylcysteine (OPMA) in the kidney. Reduction of aldehydes produced 3-HPMA, which was the principal metabolite of acrolein in urine [16], and oxidation of the aldehyde produced S-carboxyethyl-N-acetylcysteine (CEMA); the metabolic process is shown in Figure 3. A hydrophilic liquid chromatography-tandem mass spectrometry (HILIC-ESI-MS/MS) was found to be suitable for the determination of acrolein. Acrolein was non-toxic under normal conditions because of the low production and the quick degradation [20]. Polyamines were liberated from RNA once the cell was compromised, and acrolein was generated by polyamine oxidase. Polyamine oxidation products could inhibit cell proliferation and apoptosis, DNA and protein synthesis, especially spermine under the action of SMO [6]. Moreover, acrolein could disturb the self-metabolism by inhibiting two acrolein GSH conjugates metabolizing enzymes, known as alcohol dehydrogenase and aldehyde dehydrogenase [9].

## 4. The Effect of Acrolein on Neurodegenerative Diseases

Related experiments have shown that acrolein-induced oxidative damage might be related to neurodegenerative diseases [9,24]. Acrolein could induce oxidative stress [25] and interact with proteins, phospholipids [26] and DNA to form a stable Michael adduct [26,27]. Oxidative stress was regarded as the main cause of neuron loss and damage in neurodegenerative diseases [28]. The study reported significant levels of acrolein in the brain and spinal cord in patients suffering from neurodegenerative diseases (including ischemic stroke, AD, PD and MS). These neurodegenerative diseases were distinguished by increased polyamine synthesis and metabolism [29]. Polyamines, such as putrescine, spermidine, and spermine, were prevalent cellular polycations, which had a substantial influence on the optimal growth and differentiation rate of cells [30,31]. Increased polyamine metabolism led to the production of hydrogen peroxide and some reactive aldehydes associated with the death of damaged tissues [32]. Next, the mechanism of acrolein in neurodegenerative diseases will be described in detail from four aspects.

### 4.1. Ischemic Stroke

Stroke is a serious and common neurodegenerative disease due to vascular insult, involving cell injury to the CNS. Ischemic stroke, characterized by a disturbance of cerebral blood supply, including transient ischemic attacks, atherosclerotic thrombotic cerebral infarction, and cerebral embolism, is the commonest type of cerebrovascular event, accounting for approximately 85% of all strokes.

Acrolein intake may be an important factor in the induction of ischemic stroke lesion formation, such as neuronal damage, in humans. In animal experiments, reduced acrolein levels have been demonstrated to decrease infarction size and defend neurons from damage [33]. Madoka Yoshida found that the production of interleukin-6 (IL-6) and subsequently C-reactive protein (CRP) increased with the increase of acrolein in thrombosis animal models and cultured cells [34]. Acrolein induced astrocytic inflammation in a dose-dependent manner through NLRP3 inflammatory bodies, and these inflammations were modulated by ADAM10 and ascribed to p38 MAPK-activated NF-kB p65 activity [35]. JNK (c-Jun N-terminal kinase) could catalyze the phosphorylation of c-Jun and NF-kB p65, and increased JNK phosphorylation in Neuro2a-ATD cells may be a mechanism more commonly responsible for elevated GSH levels in cells, indicating that activation of JNK kinase was contributing to the increase in GSH [36]. However, the role of JNK in apoptosis was controversial, because it could not only be used as an activator of apoptosis but also in the survival pathway.

Acrolein could also be produced endogenously by lipid peroxidation in ischemic stroke [35]. Polyamines are required for the growth of eukaryotic cell and spermine is the main source of acrolein production. In neurons, there is a high concentration of polyamines, which easily appear in various pathological states of the brain and are disturbed after cerebral ischemia [30]. Acrolein was reported to elicit a malignant circle of oxidative stress that led to stroke-related neuronal damage, and the acrolein level of the plasma was elevated in the stroke patients compared to the healthy controls [37,38]. Numerous studies have found that the acrolein metabolism in stroke patients was dysregulated compared with the control group, mainly including significantly increased levels of ACR-PC, IL-6 and CRP and decreased levels of urinary 3-HPMA and plasma GSH [35,39]. It was found that inactivation of proteins by acrolein could be involved in tissue damage during brain infarction [40]. Therefore, some experiments have been performed to study the plasma proteins that could be coupled to acrolein. Yoshida found that acrolein mainly binds to Lys-557 and Lys-560 on the surface of the albumin III domain [38,40]. Saiki’s experiment found significantly lower levels of spermidine and spermidine within 24 h of stroke induction [41]. Using an in vitro stroke model of OGD cells, acrolein could induce polyamine oxidation through arginine/arginine N1-acetyltransferase (SSAT) expression caused by activation of the NF-kB pathway, which would in turn led to neuronal damage, while N-acetylcysteine was effective in preventing OGD-induced neurotoxicity by clearing acrolein [30]. In animal experiments, the activity of SMO increased with age in mice, resulting in an increase in acrolein; a decrease in GSH, which is a major intracellular acrolein detoxification compound; and a decrease in a subunit of glutathione biosynthetase. Then, the mice developed more severe cerebral infarction [42]. The study by Hideyuki Tomitori also supported the view that the increase in GSH had an important role in resistance to the neurotoxicity of acrolein in different cell types [36]. Therefore, we could assume that endogenous acrolein, as a product of a pathological state, could have a function in the pathological process and be regarded as an influential marker of stroke.

### 4.2. Alzheimer’s Disease (AD)

AD is the commonest form of dementia in the elderly, with a prevalence rate of 5% after the age of 65. The prevalence rate of people aged 85 and over has increased to approximately 30% [24]. Behavioral disorders in patients with AD were characterized by learning and memory deficits caused by neuronal death in different brain regions, including the entorhinal cortex, basal forebrain, frontal and parietal lobes, and especially the hippocampus [43]. The pathological features of the brain of patients with AD also include the deposition of amyloid-β (Aβ) in the form of fibrils surrounded by dystrophic nerve processes, forming senile plaques and intracellular neurofibrillary tangles. The structure of the medial temporal lobe is composed of a highly phosphorylated microtubule-binding protein Tau [4,44]. 

Long-term intake of acrolein could lead to mild cognitive decline and pyknosis/atrophy of hippocampal neurons, which was one of the pathological features of AD [45,46]. In hippocampal neuronal culture, acrolein had time- and concentration-dependent neurotoxicity. When the concentration was 5 mm, acrolein was more toxic than HNE [32,47]. In a dose-dependent manner, acrolein enhanced the total protein carbonylation. Proteomic analysis showed that acrolein significantly carbonylated promyosin-3 γ subtype 2, tropomyosin-5, β-actin, mitochondrial Tu translation elongation factor (EF-TUMT) and voltage-dependent anion channel (VDAC) [48]. It was discovered that acrolein causes cell death in a concentration- and time-dependent manner, increased the protein levels of amyloid precursor protein (APP), β-secretase (BACE-1) and amyloid transporter receptor of advanced glycation end products and down-regulated the levels of A-disintegrin and metalloproteinase (ADAM) 10 protein [29]. This is matching with Huang’s study [24,35]. The study of AD found that acrolein also has a certain effect on apoptosis [49]. Acrolein could lead to mitochondrial dysfunction, mainly manifested by the loss of mitochondrial transmembrane potential, the decrease in cell oxygen consumption and the decrease in ATP level [50,51].

Studies have shown that acrolein in the brain of patients with AD was significantly higher than in age-matched controls [32]. Acrolein could be produced by oxidation of Aβ, which was located in the area around early Aβ aggregates [46]. It has been found that there was a certain correlation between Aβ-induced protein oxidation and protein oxidation found in the AD brain, but there was no further research to prove it [48]. It has been reported that the ratio of protein-bound acrolein to Aβ 40/42 might be a useful biochemical indicator for subjects with mild cognitive impairment and AD [24]. Immunostaining confirmed there was acrolein in more than 50% of AD nerve fiber tangles and malnourished nerve axons around senile plaques [32]. The experiment also showed that acrolein inputs by protein were mainly present in the hippocampus of patients with AD rather than in glial cells. It was found that in the amygdala and hippocampal/parahippocampal gyrus, the average extractable acrolein in patients with AD was higher than in the control group. It has been found that in AD, acrolein increases in the brain and the increased acrolein in the brain might partially lead to brain energy loss and mitochondrial dysfunction by inhibiting the activities of the amygdala (PDH) and α-ketoglu-karate dehydrogenase (KGDH) [27,52]. Studies found that compared to age-matched controls, glutathione transferase levels in AD patients were considerably lower, such as amygdala (AMY), hippocampus/parahippocampal gyrus (HPG), inferior parietal lobule (IPL) and superior and middle temporal gyrus [53]. Glutathione transferase could detoxify acrolein and other unsaturated aldehydes, and the decrease in enzyme activity might lead to the accumulation of acrolein [32]. Studies have shown that elevated GSH in vivo can protect synaptosomal membranes from acrolein-induced protein modification of AD brain levels, which have been proven to be sites of increased oxidative stress in AD [49,54]. Thus, AD might lead to an increase in acrolein in the patient’s brain, which may be an important factor contributing to neuron degeneration.

### 4.3. Parkinson’s Disease (PD)

PD is a common neurodegenerative disease related to dyskinesia, which is characterized by both motor symptoms and non-motor symptoms [55,56]. PD is caused by dopamine (DA) deficiency leading to the degeneration of dopaminergic neurons in substantia nigra. The amino acid tyrosine can produce tyrosine hydroxylase (TH), a dopamine-limiting enzyme. In PD patients, a decrease of the TH activity in the nigrostriatal region led to lower DA levels [57]. This disease affects millions of people globally and has a serious impact on the quality of life of those who suffer from it. So far, a cure for PD has yet to be discovered. The primary function of medical management of PD was to ameliorate symptoms for an extended period of time, while minimizing adverse reactions [58]. 

Although the precise mechanisms by which acrolein injured in Parkinson’s remained poorly understood, a possible proposed model was that acrolein might play its role via oxidative stress [56]. Oxidative stress is generally recognized as one of the factors promoting the death and apoptosis of substantia nigra cells induced by acrolein and other toxic substances in patients with PD [59]. It has been proven that excessive oxidation can reduce the rate of electron transfer and cause mitochondrial dysfunction [60,61]. The mitochondrial apoptosis pathway initiated the activation of glial cells and mediated peripheral immune cells, which may eventually lead to dopaminergic cell necrosis, resulting in the aggravation of the disease [62,63]. Acrolein infused in the substantia nigra of rats could function as a parkinsonian neurotoxin with strong inhibitory effects on tyrosine hydroxylase (TH) and transporter levels in nigrostriatal DA infusion. Similar results were found in TH positive neurons and striatum DA [64]. Hence, the intake of acrolein might be contributed to the generation and development of PD lesions to some extent.

Many studies have shown that a-synuclein (aSyn) is the main component of the characteristic “Lewy body” in the brain of patients with PD [55]. The aSyn was formed by the specific binding of acrolein and protein and highly expressed in the injected substantia nigra [65]. As a rich neuronal protein, aSyn gene mutation is related to some forms of familial PD. Studies have found that acrolein can directly modify oligomeric aSyn in vitro [57,66]. A high concentration of acrolein induced the aggregation of aSyn, which led to dopaminergic imbalance, enhancement of acrolein/aSyn interaction [57], and a decrease of striatal dopamine (STR) supply in the same brain region [67,68,69]. In addition, after acrolein treatment, the nigra-striatal pathway and the primary DA synthesis pathway were activated and maladjusted, respectively, which could change the TH phosphorylation site in the whole brain [70] and decrease the activity of complex I (the main component of the electron transport chain) in the substantia nigra and frontal cortex of PD patients [57]. Therefore, acrolein produced in the patient’s brain could be considered as an influential marker of PD.

### 4.4. Multiple Sclerosis (MS)

MS is an autoimmune disease of the CNS, characterized by extensive myelin damage along the white matter bundle caused by an inappropriate inflammatory response and oxidative stress [7,71]. 

Myelin is a prominent part of the nervous system, which promotes the effective transmission of electrical signals, thereby promoting communication between the central and peripheral nervous systems and their innervated organ systems throughout the body. The structural integrity of myelin sheath mattered to the normal function of the CNS [72]. Loss of myelin and subsequent imbalance of ion channel expression causing failure of action potential conduction were considered to be the main factors of symptoms observed in patients with MS [73].

As a by-product of oxidative stress, acrolein has received widespread attention. Quite a few studies have shown that acrolein is a critical pathological factor in experimental autoimmune encephalomyelitis (EAE), which is a mouse model of MS [73,74]. The pathological effect of acrolein in EAE can be chiefly attributable to its ability to assault various biomolecules, including lipids and proteins, which are the major components of the myelin sheath and axonal membrane [73,75]. It has been found that acrolein induced demyelination by calcium non-dependent and dependent mechanisms or influencing glutamate uptake, facilitating excitotoxicity [76]. Moreover, the axon membrane was increasingly vulnerable to acrolein damage because of the loss of melin, which resulted in axonal degeneration and permanent conduction defects [74]. This might help to explain the situation in which acrolein played a role in the process of MS lesions.

## 5. Protection

Reactive carbonyl species (RCS) are good markers of oxidative stress in cells, with acrolein being one of the most toxic RCS [77]. On account of the oxidative stress of acrolein, antioxidants turn into prominent means for the treatment of acrolein poisoning. In many animal models of disease, clearance of RCS significantly alleviated or prevented disease, suggesting that clearance of RCS by antioxidants may be a potential therapeutic approach for neurodegenerative diseases [78]. Next, we will introduce antioxidants that can treat acrolein poisoning from two aspects.

### 5.1. Naturally Extracted Antioxidants

The extraction of ketones, phenols, and polysaccharides from natural products has become a research focus. Research has revealed that food extracts containing flavonoids or flavonols may inhibit the formation of acrolein during food processing in the food industry [79]. Increasing the -OH group on the B ring could enhance the protection of flavonol against acrolein among which myricetin was the most active flavonol [80]. Many studies have found that anthocyanin and its main component, anthocyanin-3-glucoside (C3G), could play a role in the prevention and treatment of central nervous system disorders by the inhibition of NO production, oxidative stress, and neuroinflammation. The neuroprotective effects of anthocyanins and C3G might also be mediated through the inhibition of JNK activation, improvement of cell degeneration, activation of brain-derived neurotrophic factor (BDNF) signal, and restoration of Ca^2+^ and Zn^2+^ homeostasis, among other mechanisms [81,82]. Additionally, Pycnogenol (PYC) could inhibit the activation of ROS and caspase-3, weaken the degree of DNA fragmentation and PARP cleavage, and finally down-regulate the degree of apoptosis. Further studies found that acrolein could activate inducible nitric oxide synthase (iNOS) to damage NADPH oxidase and damage proteins, and PYC could repair this damage by reducing the production of free radicals. Interestingly, PYC could also reduce the depletion of GSH and the toxic effect of acrolein on cells [83,84,85,86]. Other studies have found that baicalein protected against neurology by inhibiting oxidative stress, protein binding, and inflammation and by reducing acrolein-induced programmed necrosis, such as apoptosis [5]. A curcumin analogue, 1meme 5-bis (2-trifluoromethylphenyl)-1Magneto 4-pentadiene-3-one (C3), was synthesized in an experiment. It was found that the substance completely protected the oxidative damage induced by acrolein and maintained the level of GSH and the function of mitochondria. The inducing effect of C3 on Nrf2 nuclear translocation and Nrf2 target gene transcription is similar to that of curcumin. It was speculated that curcumin and C3 activate II phase enzymes by directly interfering with Nrf2/Keap1 complex to promote Nrf2 nuclear translocation [87].

Studies have also found that some food extracts containing polyphenols and polysaccharides might have inhibitory effects on acrolein [88]. Phloretin (an apple phenolic compound) had a protective effect on amino acids, proteins and cell models stimulated by acrolein, which might be achieved by inhibiting the increase of cellular protein carbonyl levels [89]. Suabjakyong’s observations indicated that the polyphenols of Phellinus Igniarius, as a substance that scavenged pathogenic acrolein, significantly reduced the infarct volume in artificially induced ischemic strokes [90]. In addition, Salicylic acid protected against acrolein-induced protein adducts, oxidative stress, structure and membranes by binding and neutralizing acrolein and acrolein protein adducts. Gu found that squid ink polysaccharide (SIP) attenuated the destruction of redox balance by inhibiting ACR-induced autophagy and apoptosis regulated by PI3K/Akt and p38 MAPK signaling pathways [91].

### 5.2. Chemically Synthesized Antioxidants and Others

Many experiments have shown that some hydrazine derivatives, such as phenylhydrazine, had a strong ability to scavenge acrolein, probably due to the use of hydrazine groups to capture acrolein [92]. The use of Hydralazine, an acrolein scavenger, was effective in reducing acrolein-induced neuronal death. The acrolein scavenger phenelzine has been shown to reduce allergic pain from inhaled acrolein.

Other methods have been shown to effectively reduce the toxicity of acrolein. It was found in a study that N-acetylcysteine (NAC) treatment could block the expression of tumor necrosis factor and inducible nitric oxide synthase induced by ischemia/reperfusion. It was suggested that pre-administration of NAC could reduce cerebral ischemia and reperfusion injury in the model of cerebral ischemia. This protective effect might be related to the inhibition of TNF-α and iNOS [20,29,93]. In addition, after spinal cord contusion in rats, intraperitoneal injection of dimercaprol could significantly reduce the content of acrolein in spinal cord tissue, because dimercaprol could bind not only to the carbon double bond of acrolein but also to the aldehyde group of acrolein [94]. It has been proven for the first time that the up-regulation of GSH mediated by the synthetic triterpenoid 2-cyano-3,12-dixooleana-1,9-dien-28-imidazolide (CDDO-Im) was the main mechanism against acrolein-induced neurotoxicity [66]. 2-aminomethylphenols could also reduce the toxicity of acrolein, although the effect is relatively insignificant. 2-mercaptoethanesulfonate (MESNA) is a minor molecule that can bind acrolein through its thiol group and has been detected in human urine to potently scavenge acrolein dose-dependently [95]. Histidine-containing scavengers such as carnosine and homocarnosine are also effective in scavenging RCS of which acrolein-creatine adducts detected in urine are among the most abundant acrolein metabolites [96].

## 6. Future Perspectives

### 6.1. Optimization of Food Thermal Processing to Reduce Acrolein Production

The thermal processing of food products is an important source of acrolein in the atmosphere. Acrolein is produced by various pathways during the thermal processing of foods and is widely distributed in fried foods, baked goods, overheated vegetable oils, alcoholic beverages, and foods rich in lipids and carbohydrates. The results of epidemiological studies suggested that the high incidence of lung cancer in Chinese women was associated with acrolein, which was produced at high temperatures from ingredients in the wok [1]. Therefore, people are exposed to acrolein through their diet, and the integration of artificial and optimized diets may be an essential way to effectively control the human intake of acrolein in food, which is important to safeguard human health. Studies have shown that excessive temperature is an important factor in the formation of acrolein in hot fats and oils. Researchers have observed that the amount of acrolein in fats and oils increased with time and temperature [1]. Consequently, optimizing the thermal treatment process of food in the diet, for example, reducing the production of acrolein by lowering the temperature during cooking, can alleviate the health problems caused by acrolein intake in humans. Of course, while reducing the generation of acrolein during food thermal processing, attention should also be paid to the maintenance and improvement of food flavor and color.

### 6.2. Exploration of More Natural Products as Food Additives to Control Acrolein Levels

Many studies have found that extracts of some natural products, such as amino acids, polyphenols, etc., could also control the formation of acrolein to a certain extent as food additives. Amino acids, which are abundant in foods, can react with acrolein under mild conditions to form adducts, aiming to reduce the production of acrolein in heat-processed foods. Free amino acids in food, such as alanine and serine, not only effectively scavenged acrolein under physiological conditions, but also rapidly and efficiently removed acrolein at high temperatures, such as 160 °C. In Addition, L-alanine has been included in the Chinese national standard (GB 2760-2014), being used in China as a flavor enhancer. In recent years, the good antioxidant activity of polyphenols has led to their widespread use in the making of various baked goods, with the aim of reducing the content of foodborne toxins and enhancing their functional properties. It was found that myricetin could scavenge acrolein produced during cookie making, demonstrating that the addition of flavonoids to baked goods could potentially inhibit the production of acrolein during food processing [80]. The catechins in matcha powder were able to significantly inhibit the accumulation of reactive carbonyl species (RCS) during baking, whose thermal stability showed the ability of matcha to be used as a food additive. Therefore, adding matcha powder to the cake dough not only added flavor to the cake but also reduced the content of RCS compounds, including acrolein [77].

In addition, amino acids and polyphenols as food additives require attention to the following issues: (1) The bioavailability of amino acids and polyphenols in humans and the risks associated with their accumulation; (2) The influence of the thermal degradation properties of polyphenols on their effectiveness in scavenging acrolein; (3) The safety of adducts produced by the reaction of amino acids and polyphenols with acrolein, and the exposure levels in different foods; (4) The interaction of amino acids and polyphenols with other components of the food; and (5) The absorption and metabolism of adducts in the human body, and so on. Therefore, it is indispensable to fully evaluate the consequences of the inclusion of amino acids and polyphenols in foods.

In conclusion, in the future food industry, it is an important development direction to discover more natural products that can be used to control foodborne toxicants or those rich in antioxidant activity, which can be used as additives not only to increase the flavor of food but also to improve the functional properties of food, control the content of foodborne toxicants, and produce food that is more in line with human health.

## 7. Conclusions

Numerous studies conducted in vitro and in vivo have shown a strong association of acrolein with neurodegenerative diseases such as ischemic stroke, AD, PD and MS. Exogenous acrolein can trigger and accelerate lesion formation through various factors (e.g., oxidative stress), while endogenous acrolein produced by the disease can also promote lesions to some extent. Of course, reports regarding the causal relationship between acrolein and disease in human epidemiological studies are currently very limited, and more advanced techniques and clinical evidence are needed to further explain and demonstrate this relationship. Several studies have reported effective scavenging of acrolein in certain matrices, such as food, but the effectiveness of these scavengers in preventing the detrimental effects of acrolein remains to be explored. Thus, acrolein may contribute to the pathogenesis of neurodegenerative diseases, and it has the potential to be a new target for the efficient usage of acrolein scavengers to improve patient symptoms in the long term.

## Figures and Tables

**Table 1:** Daily acrolein consumption from food.

Food	Acrolein Content (mg/kg or mg/L)	Daily Consumption	
		Food (g or mL/day)	Acrolein (μg/day)
Fruits	0.05	337	15–17
Vegetable oil	2.80 × 10^−3^–10.20 × 10^−3^	50	0.14–0.51
Vegetables	0.50	260–500	200–250
Potatoes	0.60	250	150
Oil	0.20	50	10
Frying fats and oils	0.276	50	13.8
Fried fish coating	0.10	NA	
Cheese	1.00	40	40
French fries	1.97–4.85	NA	
Donuts	0.90	60–400	54–360
Codfish fillet	0.10	100	10
Alcoholic beverages	0.247	84–493	20.74–121.77
Wine	3.80	43–400	163–1520
Brandy/cognac	1.50	3	2–33
Lager beer	0.002	142	0.2
Tequila	0.404	NA	
whiskey	0.252	Up to 180	Up to 45.36

NA, Data not Available.

## Data Availability

Data are contained within the article.

## Abbreviations

2-cyano-3,12-dixooleana-1,9-dien-28-imidazolide (CDDO-Im); 3-hydroxypropyl mercaptan acid (3-HPMA); α-ketoglu-karate dehydrogenase (KGDH); A-disintegrin and metalloproteinase (ADAM); A-synuclein (aSyn); Acceptable Daily Intake (ADI); Acetyl polyamine oxidase (AcPAO); Acrolein-Conjugated Protein (ACR-PC); Alzheimer’s Disease (AD); Amyloid Precursor Protein (APP); Central Nervous System (CNS); C-reactive protein (CRP); Environmental Protection Agency (EPA); Experimental Autoimmune Encephalomyelitis (EAE); Glutathione (GSH); Inducible Nitric Oxide Synthase (iNOS); Interleukin-6 (IL-6); Interleukin-8 (IL-8); JNK (c-Jun N-terminal kinase); Lipid peroxidation (LPO); Multiple Sclerosis (MS); N-acetylcysteine (NAC); Oxygen and glucose deprivation (OGD); Parkinson’s Disease (PD); produce polyunsaturated fatty acids (PUFAs); Reactive carbonyl species (RCS); S-(3-oxopropyl)-N-acetylcysteine (OPMA); S-carboxyethyl-N-acetylcysteine (CEMA); spermine oxidase (SMO); spermidine/spermine N1-acetyltransferase (SSAT); Tyrosine Hydroxylase (TH); voltage-dependent anion channel (VDAC); World Health Organization (WHO).
